# Comparison of three-dimensional soft-tissue evaluations between skeletal and pseudo-class III malocclusions

**DOI:** 10.1038/s41598-020-71772-7

**Published:** 2020-09-07

**Authors:** Burak Kale, Muhammed Hilmi Buyukcavus

**Affiliations:** 1 Department of Orthodontics, Faculty of Dentistry, Antalya Bilim University, Antalya, Turkey; 2 Department of Orthodontics, Faculty of Dentistry, Suleyman Demirel University, Isparta, Turkey

**Keywords:** Paediatric research, Musculoskeletal system, Scientific data, Mathematics and computing, Information technology

## Abstract

The aim of our study was to compare soft tissue measurements with 3D imaging methods in individuals with untreated skeletal and pseudo-Class III malocclusions. The study sample consisted of 75 patients (38 males, 37 females, mean age 12.41 ± 2.35 years) with pseudo- and true skeletal Class III malocclusions and skeletal Class I malocclusions. Soft tissue evaluations of all patients were performed using 3D stereophotogrammetric facial images. In our study, 26 landmarks, 17 linear measurements, 13 angular measurements, and 5 volume measurements were made using the 3dMD Vultus software. The significance was determined to be *p* < 0.05 in ANOVA, Tukey tests. No significant differences were found among the groups in terms of demographic data (*p* > 0.05). The skeletal Class I control group had a significantly more extended upper lip and vermillion length as compared to the Class III groups. The soft tissue convexity angle and upper nasal angle were found to be wider in the Class III malocclusion group compared to those in the Class I control group. While the pseudo-Class III group had a significantly lower midface volume, chin volume was significantly higher in the skeletal class group. Upper lip volume was significantly higher in the Class I group. Using 3dMD for guiding clinicians in the differential soft and hard tissue diagnosis of pseudo-Class III malocclusions, differences were revealed in Class I patients in the middle part of the face. In the differential diagnosis of true Class III malocclusions, chin volume was found to be different from that of Class I patients.

## Introduction

The diagnosis of Class III malocclusions is based on indications of a craniofacial deficiency between the mandible and maxilla to the cranial base^1,2^. The prevalence of malocclusions varies widely among geographic regions and ethnic groups, with ranges of 1–4% in Caucasians, 4–12% in Chinese, 9–19% in Koreans and 6% in the Swedish population^3,4^.

The etiology and features of Class III malocclusions have been well-reported as potentially having both skeletal and dental components^5,6^. Skeletal components of malocclusions are mandible prognatia, maxillar retrognatia, or a combination of both conditions. Dentoalveolar components are proclined upper incisors and retroclined lower incisors^7^. It is necessary to provide appropriate treatment planning considering differences the causes of Class III malocclusions. Skeletal (true) Class III malocclusion characteristics are deficient maxilla with a large and prognathic mandible, whereas pseudo-Class III malocclusion characteristics are deficient maxilla with a normal mandible that is caused by premature contact with functional mandible forward positioning^8^. Several scholars have reported that premature contact of the incisors increases the inclination of the upper incisors and decreases the inclination of the lower incisors in pseudo-Class III malocclusions^9–12^. Nakasima et al.^13^ examined craniofacial morphologies in 11 malocclusion types and reported that pseudo-Class III malocclusions had dental, functional, and skeletal etiological factors.

The analysis of detailed diagnosis and treatment planning in Class III malocclusions is important for clinicians in addressing disharmonious development, timing, skeletal growth, and dental patterns^14^. Clinically, for the differential diagnosis of these two types of Class III malocclusions, it is beneficial to include cephalometric evaluation of the position of the jaws and teeth according to the skeletal base, a positive response to the De Nevreze maneuver, and examination of the premature contacts in the mouth^15^. In addition, treatment plans for these skeletal Class III malocclusion types are completely different from one another. Since pseudo-Class III malocclusions can be caused by maxillary retrognathia, maxillary protractions are performed with a face mask when treating adolescents. If it is welded with premature contact or if there is an anterior inclined plane, other treatment options are available such as those involving removable appliances with a spring, as well as slow expansion appliances, among others^16^. In true Class III cases originating from mandibular prognathia, chin cap treatments are used to limit mandibular growth. Any error in diagnosis completely changes the treatment plan. It is difficult for clinicians to distinguish whether patients with a Class III malocclusion have a true Class III or a pseudo-Class III, as their profile appearance is similar^6,8,9^.

In diagnosing malocclusions, clinical photography is an essential part of orthodontic treatment planning. In recent years, photographic technology has increased to the point that it is now possible to measure facial soft tissues in orthodontics. Three-dimensional (3D) imaging methods, which are non-invasive, have become a more common and useful alternative method to traditional orthodontic 2D imaging diagnostic tools^17^. 3D imaging provides for the reconstruction of facial soft tissue models of subjects at x, y, and z coordinates, to evaluate facial distances, areas, and volumes which can be used for shape analysis and the diagnosis of complex craniofacial disorders^18^. Many studies in the literature have shown that the 3dMD face system is the right tool for linear and surface measurements with potentially wide applications in orthodontics, surgical treatment planning, and treatment evaluation. In many studies, it has been reported that this system gives more accurate results in terms of reliability and reproducibility compared to direct anthropometric measurements and 2D facial photographs^19–21^.

As a result of advances in technology, 3D imaging has become routine in orthodontic practice. The aim of our study was to compare soft tissue measurements with 3D imaging in individuals with untreated skeletal and pseudo-Class III malocclusions. The clinical significance of the results speak to clinicians’ interests in both linear and 3D measurements performed with the non-invasive, radiation-free, 3dMD face system in addition to the clinical methods used in the differential diagnosis of subgroups of Class III malocclusions. The null hypothesis tested in this study was that soft tissue measurements are not different between skeletal and pseudo-Class III malocclusions and Class I malocclusions.

## Materials and methods

This single-center, retrospective study involved 75 pre-orthodontic treatment patients (38 males, 37 females, mean age 12.41 ± 2.35 years) who received treatment at Süleyman Demirel University, Department of Orthodontics. The study was approved by the Clinical Research Ethics Committee, Süleyman Demirel University Faculty of Medicine, Isparta, Turkey (28.11.2019/297). The research was performed in accordance with the Helsinki Declaration principles and relevant guidelines and regulations. Informed consent was obtained from a parent and/or legal guardian of all subjects, as each was younger than the age of 18.

The sample size was calculated with 0.05 as the significance level and 85% power with a sample size of 24 per group. Patients who applied to Süleyman Demirel University, Faculty of Dentistry, Orthodontics Department between June 2018 and December 2019 for orthodontic treatment were evaluated according to the inclusion criteria.

The inclusion criteria were the following: (1) normal SNA angle (82° ± 2), increased SNB angle (> 82°), and negative ANB angle (< 0°) values; (2) negative Wits value; (3) normal vertical growth pattern (26° < SN/GoGn < 38°); (4) family history of mandibular prognathism for true (skeletal and dental) Class III malocclusions; (5) at least 2 incisors with negative overjet and overbite; (6) centric relation-centric occlusion discrepancy (the information filled by the physicians in the anamnesis forms of the patients were used as reference); (7) no previous orthodontic treatment; (8) Cervical Vertebral Maturation (CVM) between stage 2 and 3 at initial records; and (9) identifiable landmarks on all of the 3D images. Those with a history of orthodontic treatment, craniofacial syndromes, and/or different malocclusions were excluded.

Forty-nine individuals who met the inclusion criteria from a pool of 437 patients who had 3dMD images at the beginning of the treatment were included in the pseudo- and true Class III groups. In addition, 26 patients with a skeletal Class I malocclusion and normal vertical growth pattern were also included in the Class I malocclusion group as the control group. The Class III groups were compared to the Class I control group. Participation consent was obtained from patients and parents included in the study, which allowed for their data to be used in scientific publications. To determine skeletal anomalies and vertical growth patterns, lateral cephalometric radiographs of patients were evaluated before treatment. Both ANB angle and Wits values were evaluated to design the skeletal classification. Consequently, the study sample consisted of 75 patients ages 8–14 years old with a pseudo- or true skeletal Class III malocclusion or a skeletal Class I malocclusion.

### Data analysis

Soft tissue evaluations of all patients were performed using 3D stereophotogrammetric facial images. In this study, 3D facial images were taken using the 3dMDface System (3dMD LLC, Atlanta, GA, USA/https://www.3dmd.com/). The system is used by combining the structured light system and stereophotogrammetry technology with a synchronized, multi-camera configuration^22^. Images were taken in standard office lighting conditions and in the natural head position with relaxed lips, which has high clinical reproducibility^23^. The device was calibrated according to the manufacturer’s instructions before images of each patient were captured.

Soft tissue analysis was performed using 3dMD VultusR software (3dMD VultusR software version 2.3.0.2, 3dMD, Atlanta, GA, USA/https://www.3dmd.com/). All of the 3D facial images were reoriented for standardization and all the sections of the images that were not included in the analyses were cut off so as to have similarity among subjects.

**Figure 1:**
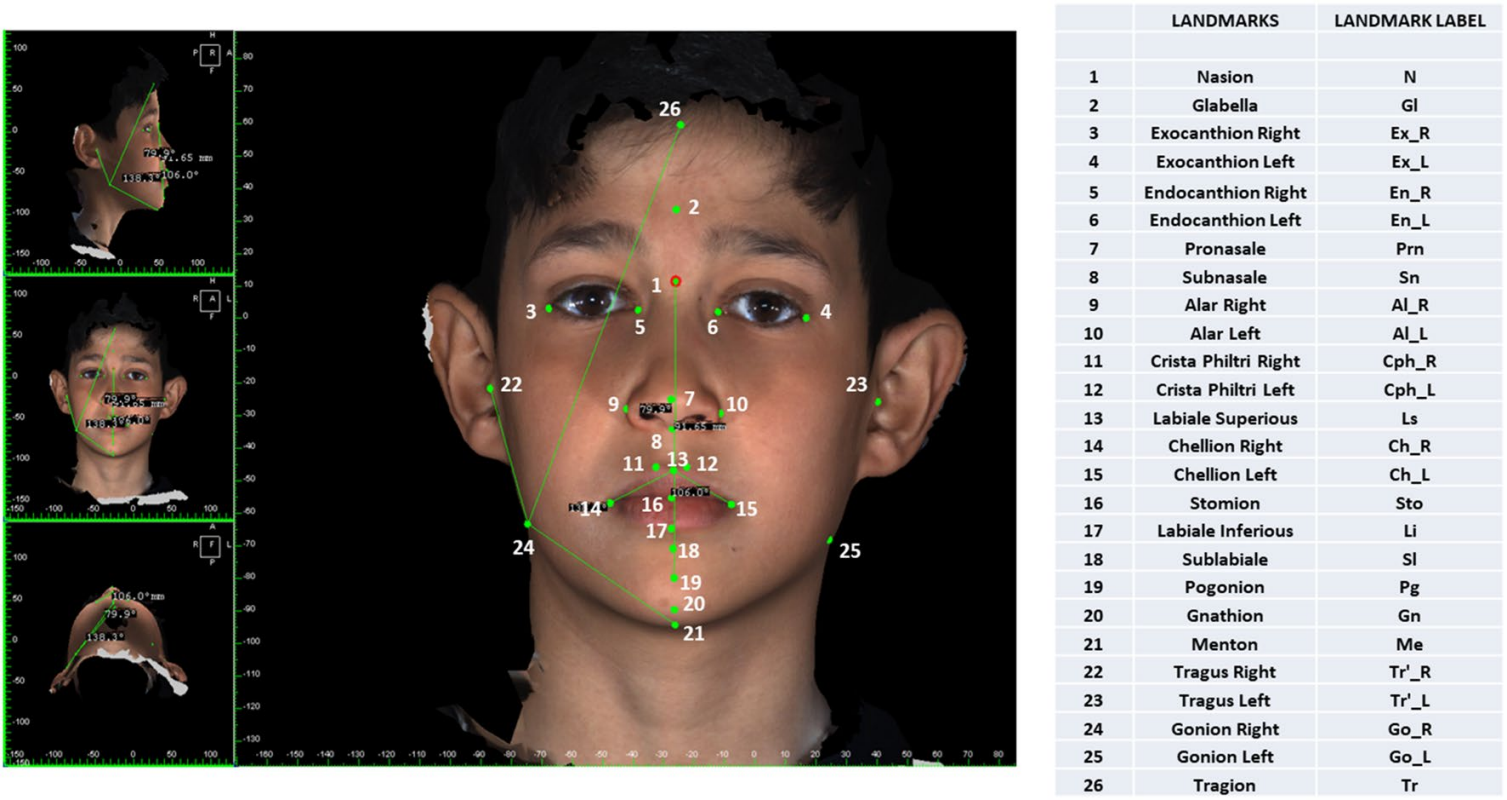
Landmarks used in 3D facial soft-tissue evaluation.

**Figure 2:**
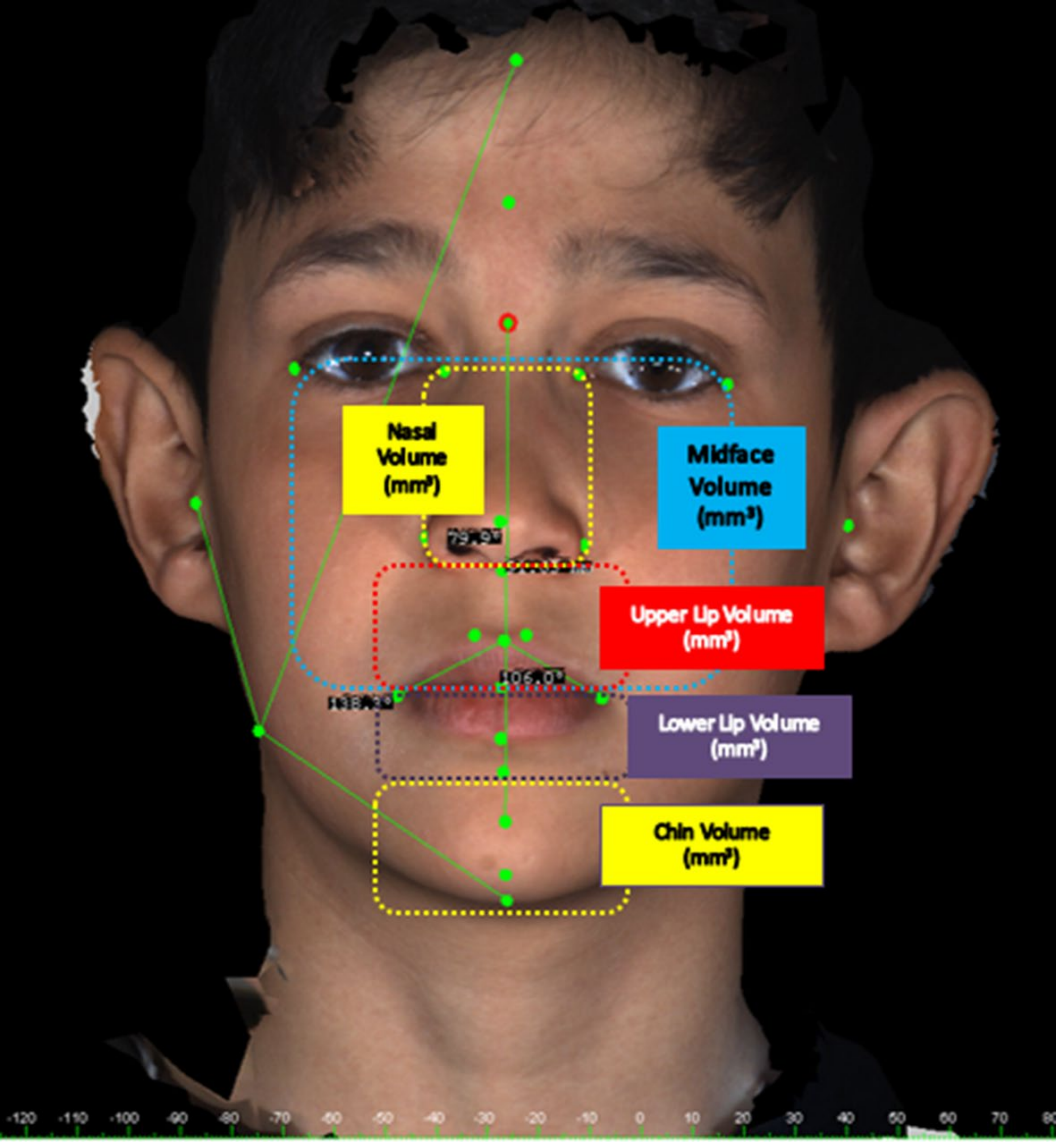
3D volume measurements used in 3dMD Face System.

The landmarks used in our study are shown in Fig. 1: 26 landmarks and a total of 17 linear, 13 angular, and 5 volume measurements (Fig. 2) were made with the 3dMD Vultus software. The facial (15), nasal (5), lip (10), and volume (5) measurements are shown in Table 1. In the linear measurements made from the profile, the average of the measurements of the right and left sides was taken as a single value in all groups.

**Table 1:** Measurements and landmarks used in 3D facial soft-tissue evaluation.

Measurements	Landmarks
**Facıal measurements**	
Total face height	N–Pg
Upper anterior face height	N–Sn
Lower anterior face height	Sn–Pg
Upper face depth	N–Tr
Middle face depth	Sn–Tr
Lower face depth	Pg–Tr
Mandibular height	Gn–Sl
Bigonial width	GoR–GoL
Soft tissue convexity angle	N–Sn–Pg
Total face convexity angle	Gl–Prn–Pg
H angle	N–Pg–Ls
SNA	Tr–N–Sn
SNB	Tr–N–Sl
ANB	Sn–N–Sl
Gonial angle	Tr–Go–Me
**Nasal measurements**	
Alar base width	AlR–AlL
Nasal tip protrusion	Sn–Prn
Nasal tip protrusion angle	AlR–Prn–AlL
Nasal bridge length	N–Prn
Upper nasal angle	N–Prn–Sn
**Lıp measurements**	
Upper lip length	Sn–Sto
Upper lip angle	Sn–Ls–Sto
Upper lip vermillion length	Ls–Sto
Lower lip length	Sl–Sto
Lower lip angle	Sto–Li–Sl
Lower lip vermillion length	Li–Sto
Philtrum width	CphR–CphL
Lip width	ChR–ChL
Nasolabial angle	Prn–Sn–Ls
Labiomental angle	Sl–Li–Pg
**Volume measurements**	
Midface volume (mm^3^)	ExR–ExL/ChR–ChL
Nasal volume (mm^3^)	AlR–AlL/EnR–EnL
Upper lip volume (mm^3^)	Sn/ChR–ChL
Lower lip volume (mm^3^)	Sl/ChR–ChL
Chin volume (mm^3^)	Sl–Me/ChR–ChL

For volume measurements, sagittal, axial, coronal, and 3D images were recorded in STL format. The STLs were transferred to a network computer workstation, where soft tissue volumetric analysis was performed using MIMICS 20.0 software launched by Materialized (Materialize Europe, World Headquarters, Leuven, Belgium/https://www.materialise.com/en). The volume of individual study regions was calculated using Mimics Software. In addition, since the soft tissue measurements may have been affected by gender differences, measurements in each group were also taken and evaluated separately for females and males.

To determine the individual error of measurement, 25 subjects were randomly selected from all groups for a second 3dMD analysis. The second set of measurements was taken 10 days after the first set. All of the second measurements were compared to the first to assess the reliability of analysis using Cronbach’s alpha reliability test (*r*). All* r* values were in the range of 0.941–1, suggesting that the first and second measurements were almost identical or had only negligible errors.

### Statistical analysis

The SPSS software package program (SPSS Inc., version 21.0; Chicago, IL, USA/https://www.ibm.com/tr-tr/products/spss-statistics) was used to perform the statistical analyses. The distributions of gender and growth development periods (CVM) of the patients were compared among pseudo-Class III, skeletal Class III, and Class I groups with the Pearson chi-square test. In addition, an independent t-test was used to compare the ages and CVM periods of males and females in each group. The Cronbach’s alpha reliability test was used for method errors, and the Shapiro–Wilk test was used to determine whether the data were normally distributed. In normally distributed data, the ANOVA test was used for testing the differences among groups. When significant differences between the groups were demonstrated, the Tukey (post hoc) test was used between paired groups. The significance was determined to be *p* < 0.05 in ANOVA, Tukey tests. In order to evaluate the differences between genders, each parameter was compared with the *t *test.

### Ethics approval and consent to participate

The study was approved by the Clinical Research Ethics Committee, Süleyman Demirel University Faculty of Medicine, Isparta, Turkey (Ethics approval decision number: 28.11.2019/297). Consent to publish was obtained from patients and parents. (Written consent is obtained from patients who apply to our clinic for treatment purposes, indicating that their radiographs, 3dMD images or materials can be used scientific articles.)

## Results

The mean age of the subjects was 12.41 ± 2.35 years (38 females; 37 males). The mean age of the skeletal Class III group was 12.45 ± 2.15 years (13 females, 12 males; minimum 8.27, maximum 14.11); for the pseudo Class III group it was 12.09 ± 2.89 years (12 females, 12 males; minimum 8.6, maximum 14.3); and for the skeletal Class I control group it was 12.66 ± 2.01 years (13 females, 13 males; minimum 8.57, maximum 14.33).

There was no significant difference between the malocclusion groups in terms of chronological age, gender or distribution of CVM stages [*p* > 0.05 (Table 2)]. Also, when the males and females in each group were compared, no significant difference was found in terms of chronological age or distribution of growth development periods [*p* > 0.05 (Table 2)]. These results show that the individuals in each group were well-matched in terms of age, gender, and growth development periods. Comparisons of facial measurements are shown in Table 3 and results of the nasal and lip measurements are shown in Table 4. Volume measurements are shown in Table 5.

**Table 2:** Comparison of the chronological ages, gender distributions, and growth development periods between the groups.

	Gender distribution (Male/Female)	Chronological age (years) Mean ± SD	Growth development period
Group 1 Skeletal Class III group	12 male	11.33 ± 2.06	CS 2 (n = 7, 58%) CS 3 (n = 5, 42%)
	13 female	13.62 ± 1.23	CS 2 (n = 6, 46%) CS 3 (n = 7, 54%)
	N = 25	12.45 ± 2.15	CS 2 (n = 13, 52%) CS 3 (n = 12, 48%)
P′	–	NS^c^	NS^a^
Group 2 Pseudo Class III group	12 male	11.25 ± 1.89	CS 2 (n = 7, 58%) CS 3 (n = 5, 42%)
	12 female	12.93 ± 2.01	CS 2 (n = 7, 58%) CS 3 (n = 5, 42%)
	N = 24	12.09 ± 2.89	CS 2 (n = 14, 58.33%) CS 3 (n = 10, 41.67%)
P′	–	NS^c^	NS^a^
Group 3 Skeletal Class I control group	13 male	12.45 ± 1.58	CS 2 (n = 7, 53%) CS 3 (n = 6, 47%)
	13 female	12.87 ± 1.93	CS 2 (n = 6, 47%) CS 3 (n = 7, 53%)
	N = 26	12.66 ± 2.01	CS 2 (n = 13, 50%) CS 3 (n = 13, 50%)
P′	–	NS^c^	NS^a^
P	.852^a^	.922^b^	.775^a^

*SD* standard deviation, *N* number, *P* comparison of malocclusion groups, *P′* comparison of the ages and CVM periods of male and female in each group.

^a^Results of Pearson chi-square test.

^b^Results of ANOVA test.

^c^Results of independent test.

**Table 3:** Comparison of facial measurements made on soft tissues according to groups and genders.

		Group I Skeletal Class III Mean ± SD	Group II Pseudo Class III Mean ± SD	Group III Control Class I Mean ± SD	Post-hoc tests			P^†^
					1 versus 2	1 versus 3	2 versus 3	
**Facıial measurements**								
Total face height (N’–Pg’)	F	**91.18 ± 6.06**^**a**^	91.12 ± 8.16	**88.4 ± 5.91**^**a**^	NS	NS	NS	NS
	M	**94.41 ± 5.84**^**b**^	93.58 ± 6.12	**92.14 ± 2.08**^**b**^				
	T	92.73 ± 6.17	92.35 ± 7.14	90.27 ± 3.99				
Upper anterior face height (N’–Sn’)	F	50.29 ± 3.73	49.59 ± 4.88	50.61 ± 4.15	NS	NS	NS	NS
	M	52.03 ± 2.93	51.33 ± 3.78	51.93 ± 2.81				
	T	51.18 ± 3.81	50.46 ± 4.33	51.27 ± 3.48				
Lower anterior face height (Sn’–Pg’)	F	41.97 ± 4.48	42.7 ± 4.81	39.92 ± 4.55	NS	NS	NS	NS
	M	43.5 ± 3.71	43.1 ± 4.08	41.94 ± 2.63				
	T	42.7 ± 4.56	42.9 ± 4.44	40.93 ± 3.59				
Upper face depth (N’–Tr’)	F	51.74 ± 9.25	49.71 ± 10.05	51.81 ± 10.21	NS	NS	NS	NS
	M	52.93 ± 7.68	50.93 ± 8.71	52.73 ± 9.87				
	T	52.64 ± 9.41	50.32 ± 9.38	52.27 ± 10.04				
Middle face depth (Sn’–Tr’)	F	**101.87 ± 11.02 **^**a**^	**98.83 ± 12.94 **^**a**^	104.49 ± 11.29	NS	NS	**	**
	M	**104.79 ± 10.83**^**b**^	**102.41 ± 11.78**^**b**^	106.41 ± 9.62				
	T	103.64 ± 11.21	100.62 ± 12.36	105.45 ± 10.46				
Lower face depth (Pg’–Tr’)	F	**143.35 ± 6.46**^**a**^	**138.96 ± 6.85**^**a**^	139.75 ± 5.18	*	*	NS	*
	M	**147.42 ± 6.13**^**b**^	**142.96 ± 6.73**^**b**^	141.75 ± 5.34				
	T	145.83 ± 6.58	140.96 ± 6.79	140.75 ± 5.26				
Mandibular height (Gn’–Sl’)	F	20.46 ± 2.49	17.68 ± 1.94	20.26 ± 4.55	NS	NS	NS	NS
	M	21.47 ± 2.08	19.74 ± 1.42	21.24 ± 4.03				
	T	20.82 ± 2.53	18.71 ± 1.68	20.75 ± 4.29				
Bigonial width (GoR’–GoL’)	F	**105.29 ± 6.97**^**a**^	**101.97 ± 5.96**^**a**^	103.51 ± 7.34	NS	NS	NS	NS
	M	**109.23 ± 6.89**^**b**^	**105.81 ± 5.18**^**b**^	107.57 ± 6.42				
	T	107.12 ± 7.11	103.89 ± 5.57	105.54 ± 6.88				
Soft tissue convexity angle (N’–Sn’–Pg’)	F	160.65 ± 6.91	163.89 ± 5.19	156.62 ± 7.73	NS	*	*	*
	M	163.81 ± 7.47	164.01 ± 4.42	157.94 ± 6.09				
	T	163.43 ± 7.03	163.95 ± 4.81	157.28 ± 6.91				
Total face convexity angle (Gl’–Prn’–Pg’)	F	133.24 ± 5.76	136.78 ± 7.84	129.97 ± 5.25	NS	*	*	*
	M	135.93 ± 5.04	137.06 ± 7.47	130.93 ± 4.07				
	T	135.55 ± 5.86	136.92 ± 7.64	130.45 ± 4.66				
H angle (N’–Pg’–Ls’)	F	12.19 ± 4.14	13.25 ± 4.54	17.61 ± 6.34	NS	*	*	*
	M	12.65 ± 4.08	13.91 ± 4.22	18.73 ± 6.12				
	T	12.41 ± 4.22	13.58 ± 4.38	18.17 ± 6.23				
SNA’ (Tr’–N’–Sn’)	F	**171.19 ± 3.64**^**a**^	174.27 ± 3.08	175.66 ± 2.41	NS	NS	NS	NS
	M	**174.41 ± 3.62**^**b**^	173.93 ± 2.81	175.3 ± 2.85				
	T	174.15 ± 3.71	174.1 ± 3.35	175.48 ± 2.63				
SNB’ (Tr’–N’–Sl’)	F	**169.53 ± 5.42**^**a**^	169.87 ± 6.59	168.21 ± 5.23	NS	*	NS	*
	M	**172.53 ± 5.06**^**b**^	170.57 ± 6.23	169.02 ± 5.33				
	T	172.48 ± 5.52	170.22 ± 6.41	168.61 ± 5.28				
ANB (Sn’–N’–Sl’)	F	7.41 ± 3.15	7.26 ± 2.05	10.21 ± 3.57	NS	*	*	*
	M	7.52 ± 3.14	7.38 ± 2.13	10.08 ± 3.21				
	T	7.54 ± 3.21	7.32 ± 2.09	10.14 ± 3.39				
Gonial angle (Tr’–Go’–Me’)	F	**137.41 ± 5.46**^**a**^	**134.97 ± 4.32**^**a**^	138.89 ± 5.48	NS	NS	NS	NS
	M	**141.63 ± 5.75**^**b**^	**139.11 ± 3.04**^**b**^	141.05 ± 5.14				
	T	139.79 ± 5.56	137.04 ± 3.68	139.97 ± 5.31				

*F* female, *M* male, *T* total, *SD* standard deviation, *NS* not significant, *P*^*†*^: Results of ANOVA test (Post-Hoc Tukey test), *P(a,b)* results of *t* test; comparison by gender; difference was detected in the parameters expressed in different bold letters.

*p < 0.05; **p < 0.01; ***p < 0.001.

**Table 4:** Comparison of nasal and lip measurements made on facial soft tissues according to groups and genders.

		Group I Skeletal Class III Mean ± SD	Group II Pseudo Class III Mean ± SD	Group III Control Class I Mean ± SD	Post-hoc tests			P^†^
					1 versus 2	1 versus 3	2 versus 3	
**Nasal measurements**								
Alar base width (AlR’–AlL’)	F	32.82 ± 3.79	31.92 ± 2.92	30.08 ± 3.63	NS	NS	NS	NS
	M	34.42 ± 3.27	34.04 ± 2.58	33.02 ± 3.41				
	T	33.21 ± 3.96	32.98 ± 2.75	31.91 ± 3.52				
Nasal tip protrusion (Sn’–Prn’)	F	15.61 ± 1.01	14.85 ± 2.79	15.41 ± 1.75	NS	NS	NS	NS
	M	16.42 ± 0.95	16.21 ± 1.47	15.73 ± 1.31				
	T	16.29 ± 1.05	15.53 ± 2.63	15.57 ± 1.53				
Nasal tip protrusion angle (AlR’–Prn’–AlL’)	F	105.15 ± 6.37	106.94 ± 6.5	106.68 ± 4.53	NS	NS	NS	NS
	M	108.5 ± 5.71	108.1 ± 6.08	107.94 ± 4.63				
	T	107.68 ± 6.65	107.52 ± 7.09	107.31 ± 5.72				
Nasal bridge length (N’–Prn’)	F	42.04 ± 4.13	43.69 ± 4.82	43.21 ± 2.47	NS	NS	NS	NS
	M	42.93 ± 4.68	43.93 ± 4.71	45.73 ± 2.87				
	T	43.89 ± 4.32	43.81 ± 5.36	44.47 ± 3.21				
Upper nasal angle (N’–Prn’–Sn’)	F	40.02 ± 3.79	41.07 ± 1.98	39.49 ± 4.53	NS	NS	NS	NS
	M	41.79 ± 3.83	42.41 ± 1.78	40.41 ± 4.62				
	T	41.77 ± 3.96	41.74 ± 2.43	39.95 ± 5.15				
**Lip measurements**								
Upper lip length (Sn’–Sto’)	F	18.04 ± 2.42	18.22 ± 1.64	21.77 ± 2.41	NS	*	*	**
	M	19.42 ± 2.13	18.96 ± 1.73	21.95 ± 2.34				
	T	18.84 ± 2.53	18.59 ± 2.02	21.86 ± 2.91				
Upper lip angle (Sn’–Ls’–Sto’)	F	105.16 ± 7.58	105.32 ± 5.56	111.27 ± 4.68	NS	*	*	*
	M	107.47 ± 7.08	106.74 ± 6.42	111.41 ± 5.03				
	T	106.64 ± 8.26	106.03 ± 6.73	111.34 ± 5.81				
Upper lip vermillion length (Ls’–Sto’)	F	6.51 ± 1.82	6.35 ± 1.41	7.61 ± 0.58	NS	*	*	*
	M	7.23 ± 1.89	6.81 ± 1.18	8.57 ± 0.42				
	T	6.79 ± 1.90	6.58 ± 1.65	8.09 ± 1.09				
Lower lip length (Sl’–Sto’)	F	13.78 ± 1.96	14.23 ± 2.28	13.48 ± 2.31	NS	NS	NS	NS
	M	15.01 ± 1.47	15.01 ± 2.22	14.24 ± 2.09				
	T	14.39 ± 2.05	14.62 ± 2.43	13.86 ± 2.58				
Lower lip angle (Sto’–Li’–Sl’)	F	115.78 ± 6.58	120.84 ± 6.49	118.43 ± 6.52	NS	NS	NS	NS
	M	118.93 ± 5.04	124.06 ± 7.41	120.93 ± 6.02				
	T	117.73 ± 7.21	122.45 ± 6.28	119.68 ± 7.31				
Lower lip vermillion length (Li’–Sto’)	F	7.85 ± 1.54	6.19 ± 1.88	7.51 ± 2.08	NS	NS	NS	NS
	M	8.65 ± 2.08	8.91 ± 2.02	8.73 ± 2.12				
	T	8.2 ± 1.61	7.55 ± 1.67	8.12 ± 2.03				
Philtrum width (CphR’–CphL’)	F	12.44 ± 2.61	10.81 ± 1.75	11.9 ± 1.88	NS	NS	NS	NS
	M	13.41 ± 2.62	11.93 ± 1.83	11.39 ± 2.05				
	T	12.99 ± 2.73	11.37 ± 1.65	11.63 ± 2.01				
Lip width (ChR’-ChL’)	F	44.84 ± 3.76	43.41 ± 4.16	43.9 ± 3.58	NS	NS	NS	NS
	M	47.53 ± 4.06	45.57 ± 4.23	44.02 ± 4.33				
	T	46.81 ± 3.93	44.49 ± 4.01	43.96 ± 4.13				
Nasolabial angle (Prn’–Sn’–Ls’)	F	113.41 ± 12.51	116.44 ± 7.58	108.56 ± 7.61	NS	**	**	**
	M	117.52 ± 10.14	117.38 ± 8.13	109.08 ± 8.21				
	T	114.21 ± 11.39	116.91 ± 7.71	108.82 ± 9.33				
Labiomental angle (Sl’–Li’–Pg’)	F	143.76 ± 15.95	142.05 ± 12.18	143.65 ± 20.03	NS	NS	NS	NS
	M	147.63 ± 14.75	143.11 ± 13.04	142.05 ± 15.14				
	T	146.94 ± 16.65	142.58 ± 13.21	142.85 ± 20.71				

*F* female, *M* male, *T* total, *SD* standard deviation, *NS* not significant, *P*^*†*^ results of ANOVA test (Post-Hoc Tukey test), *P(a,b)* results of *t* test; comparison by gender; difference was detected in the parameters expressed in different bold letters.

*p < 0.05; **p < 0.01; ***p < 0.001.

**Table 5:** Comparison of volume measurements made on facial soft tissues according to groups and genders.

		Group I Skeletal Class III Mean ± SD	Group II Pseudo Class III Mean ± SD	Group III Control Class I Mean ± SD	Post-hoc tests			P^†^
					1 versus 2	1 versus 3	2 versus 3	
**Volume measurements**								
Midface volume (mm^3^) (ExR–ExL/ChR–ChL)	F	**28,113 ± 4,327 **^**a**^	**24,953 ± 3,042 **^**a**^	30,047 ± 3,945	NS	NS	NS	NS
	M	**29,346 ± 4,327 **^**b**^	**27,341 ± 2,958 **^**b**^	30,302 ± 5,416				
	T	28,322 ± 4,876	25,147 ± 3,306	30,143 ± 5,341				
Nasal volume (mm^3^) (AlR–AlL/EnR–EnL)	F	10,494 ± 2058	10,809 ± 1871	11,112 ± 2,416	NS	NS	NS	NS
	M	11,642 ± 2095	10,921 ± 1947	12,573 ± 2,131				
	T	10,955 ± 1958	10,865 ± 2014	11,842 ± 2,143				
Upper lip volume (mm^3^) (Sn/ChR–ChL)	F	2,629 ± 629	2,445 ± 805	3,202 ± 541	NS	NS	NS	NS
	M	2,885 ± 571	2,581 ± 608	3,294 ± 763				
	T	2,745 ± 655	2,513 ± 701	3,248 ± 845				
Lower lip volume (mm^3^) (Sl/ChR–ChL)	F	3,585 ± 719	3,389 ± 873	3,167 ± 906	NS	NS	NS	NS
	M	3,893 ± 468	3,493 ± 671	3,273 ± 871				
	T	3,743 ± 751	3,441 ± 805	3,220 ± 913				
Chin volume (mm^3^) (Sl–Me/ChR–ChL)	F	**544 ± 295 **^**a**^	467 ± 149	458 ± 171	NS	NS	NS	NS
	M	**601 ± 383 **^**b**^	491 ± 178	504 ± 162				
	T	568 ± 101	479 ± 151	481 ± 126				

*F* female, *M* male, *T* total, *SD* standard deviation, *NS* not significant, *P*^*†*^ results of ANOVA test (Post-Hoc Tukey test), *P(a,b)* results of *t* test; comparison by gender; difference was detected in the parameters expressed in different bold letters.

*p < 0.05; **p < 0.01; ***p < 0.001.

The skeletal Class I control group had a significantly more extended upper lip and vermillion length as compared with the Class III groups. In the skeletal Class III group, the soft tissue convexity angle, total facial convexity angle, upper nasal angle, nose tip protrusion angle, and philtrum width were found to be wider than those of the Class I group, while the H angle and nasolabial angle were narrower than those of the Class I control group. Compared to the control group, the nasal bridge and the upper lip length were decreased in the pseudo-Class III group. In the pseudo-Class III group, the soft tissue convexity angle and the total facial convexity angle were larger than in the control group, while the H angle was smaller than in the control group. Also in the pseudo-Class III group, the upper nasal angle and the nasal tip protrusion angle was larger than that of the control group.

When the nasal measurements were evaluated in the skeletal Class III group, the alar base width, nasal tip protrusion, nasal tip protrusion angle, and upper nasal angle were increased in comparison to those of the Class I group and the pseudo-Class III group. The skeletal Class III group had a significantly shorter upper lip vermillion length as compared to the control group. In the skeletal Class III group, while the soft tissue convexity angle, the total facial convexity angle, and the labiomental angle were larger than in the control group, the H angle, upper lip angle, and nasolabial angle were less than in the control group.

When the volume measurements were examined, there were significant differences among the groups in midface, upper lip, and chin volumes (*p* < 0.05). While midface and upper lip volumes were significantly higher in the Class I control group, chin volumes were significantly higher in the skeletal Class III group.

When the differences between the genders were examined, it was observed that there was a significant difference in midface volume, chin volume, total face height, middle face depth, lower face depth, bigonial width, SNA, SNB, and gonial angle in the skeletal Class III group. A significant difference was also observed in midface volume, middle face depth, lower face depth, bigonial width, and gonial angle in the pseudo-Class III group. In the skeletal Class I control group, the total face height measurement was found to be significantly different between males and females.

## Discussion

In order to optimally treat Class III malocclusions, it is necessary to differentiate between pseudo-Class III malocclusions and true (skeletal) Class III malocclusions. A cephalometric evaluation, De Nevreze procedure, and clinical examination can be used to distinguish between these two types of Class III malocclusions^15,16^. In this study, in addition to using the routine methods, our aim was to contribute to clinicians’ abilities to offer differential diagnoses with measurements made using the 3dMD face system, which has increased in popularity in recent years.

Genetic (age, gender, race, CVM stages, etc.) and environmental factors can affect the etiology of malocclusions^13^. Patients in a certain region (the western Mediterranean region) were included in the study to minimize changes due to racial factors. We also carefully considered the fact that the patients in our study were of a certain age. Individuals of similar age were included, since the mean age of the patients who applied to our clinic for treatment was approximately 12–13 years. However, we statistically evaluated whether there was an age difference among the groups. It was observed that the groups were matched well in terms of age.

With regard to gender evaluations, there was no significant difference between males and females with Class III malocclusions in soft tissue studies^24–29^. In our study, although soft tissue parameters were generally higher in males, a significant difference was observed in approximately one-quarter of the measurements made by gender. In skeletal and pseudo-Class III groups, mid-face measurements were significantly higher in males, while in the Class I malocclusion group, soft tissue characteristics were almost the same across the genders. Bacetti et al. found that soft tissue measurements of individuals in the CS1, CS2, CS3, and CS4 periods were similar in individuals with an untreated Class III malocclusion^30^. They observed differences only in soft tissues in the post-pubertal period. However, in our study, patients in the CS2 and CS3 periods were used to minimize soft tissue and skeletal changes during different periods of growth; these were statistically evaluated.

Soft tissue analysis is one of the most important types of analysis for diagnosis and treatment planning in orthodontics as well as for the evaluation of treatment results. Until now, soft tissue analysis was performed on the photos taken at the beginning of the treatment and on the lateral cephalometric radiographs. However, the fact that both photographs and radiographs were in two dimensions restricted the evaluations and did not provide detailed analysis. 3D digital imaging techniques have gained popularity in recent years because they eliminate these difficulties^31^.

The stereophotogrammetry technique has been recognized as the most promising among all 3D soft tissue imaging methods. This technique works on the principle that two or more cameras simultaneously capture images from different angles on an object and create a 3D image of soft tissue morphology with the help of special computer software^32^.

The accuracy of measurements in orthodontics and craniofacial surgery is critical in diagnosis and treatment planning. With advances in technology, 3D imaging devices have increased the accuracy of measurements made by clinicians as part of the treatment planning and evaluation process^33^. In addition, orthodontists can evaluate all angular, linear, and volumetric relationships in soft tissues (face, nose, lips, cheeks, chin, etc.) and analyze and evaluate vertical and transverse facial asymmetries. For the assessment of the soft tissue profile, previous studies of Class III patients involved the use of the 2D methodology (cephalometric radiographs), which carries radiation risks and is therefore inappropriate for adolescents. We first described the soft tissue profile in different types of adolescent patients with Class III diagnoses, using the non-invasive, fast, easy, and reliable 3D methodology described earlier in the literature, which could become an important diagnostic tool in the future.

Since there has not been a similar, previous study, we chose to compare our results to those from studies whose authors used cephalometric analysis. In our study, patients with a normal vertical growth pattern (normodivergent) were selected by examining lateral cephalometric films; there were no significant differences among the three groups in terms of facial heights.

According to our results, statistically significant differences in the soft tissue measurements of Class I and pseudo-Class III malocclusions were detected in measurements related to the maxilla. In the pseudo-Class III group, mid-face depth was lower than in the other two groups due to maxillary retrognathia. In addition, parameters related to the upper lip (upper lip length, upper lip angle, and upper lip vermillion length) were found to be less in the pseudo-Class III group in direct proportion to the maxillary deficiency.

The movements of skeletal structures and the soft tissues surrounding them were not exactly the same. Apart from the position of the maxilla in the movement of the upper lip, other factors such as muscle contraction, muscle tone, and the position of the teeth were also effective. Therefore, differences in the angle and length of the upper lip were also effective for the maxillary to be retrognathic, but the results cannot solely be linked to the maxilla position. The soft tissue profile of patients with maxillary retrognathia has been studied cephalometrically in many studies. In the literature, malocclusions have usually been compared with each other and many of them have involved the use of cephalometric radiographs.

Singh et al.^34^ found that most of the parameters they examined were statistically different in their study in which they compared the soft tissue properties of patients with Class III and Class I malocclusions. Chang et al.^35^ also reported that the developmental deficiency of the nasomaxillary complex in skeletal Class III patients resulted in a mid-face retrognathic appearance. Rabie and Gu^36^ also stated that the main characteristic features of pseudo-Class III malocclusions are the retrusive upper lip.

Patients with skeletal Class III malocclusions seem to have moderate hypoplasia due to the prognathic mandible, while patients with pseudo-Class III malocclusions seem to be prognathic due to maxillary retrognathia. This situation may mislead clinicians in the treatment of these two types of Class III malocclusions, whose treatment protocols are different. In this study, in which we compared the soft tissue parameters in three dimensions, we aimed to determine the correct parameters that clinicians should look at in order to help them plan their treatment and make a more accurate diagnosis. Our findings showed that patients with a true skeletal Class III malocclusion had larger mandibles, prominent lower lips, and pogonions. Individuals with a true skeletal Class III malocclusion were noted to have lower face depth, mandibular height, bigonial width, lower lip, vermilion and philtrum widths, and labiomental angle. However, these differences were not statistically significant.

It has been reported in the literature that patients with a skeletal Class III malocclusion have a more horizontal growth pattern in their mandible. In the 3dMD images in our study, it was found that the soft tissues of the mandible and its surrounding area were more prominent and prognathic in skeletal Class III patients, which is consistent with the literature^6,37,38^. The results of this study showed that there was a distinct lower lip in the true skeletal Class III group, although it was statistically insignificant.

When we evaluated 3D volume measurements, which is the most important advantage of the 3dMD technique, significant differences were detected between the groups in midface, upper lip, and chin volume. Patients in the pseudo-Class III group were found to be close to maxillary retrognathia in light of our measurements. This is because pseudo- and true Class III malocclusions are intertwined with each other and they might have transitions in time. Therefore, in the pseudo-Class III group, both linear and volume measurements on the midface were found to be significantly less. Chin volume was significantly higher in the skeletal Class III group. Chin volume can be used as a parameter for differential diagnosis of true Class III malocclusions. Upper lip volumes were significantly higher in the Class I group than in either of the Class III groups. It can be concluded that the upper lip volume is also a parameter that can be used to distinguish pseudo-Class III malocclusions from Class I malocclusions. Upper lip volume was lower in both true and pseudo-Class III groups. The reason for this may be the lower upper lip volume due to maxillary retrognathia, or if there is a pseudo-Class III condition due to upper incisor retrusion in this group, the upper lip volume may be less in the pseudo-Class III group due to the upper incisor position.

These results are consistent with the findings of other studies in the literature. The soft tissue data obtained from our study revealed that the structural features of soft tissues in patients with skeletal Class III malocclusions may be an important factor in the diagnosis of a malocclusion. Kasai stated in his study that there is a strong but complex relationship between skeletal and soft tissue changes caused by Class III malocclusions; this supports our results.

Clinicians consider correction of the concave face profile as a mandatory target with orthopedic treatments in patients with a Class III malocclusion. Our study has shown that the mid-face and upper lip region can be evaluated in the differential diagnosis of pseudo-Class III patients. According to our findings, pseudo-Class III patients had more retrognathic mid- face and upper lip structure than Class I patients. Recent findings from the literature involving facial analyses of children have shown that children with a Class III malocclusion typically have a concave facial profile, maxillary deficiency, prognathic mandible, and retrusive midface in comparison to patients with a Class I malocclusion; those results support our findings^38^.

Also in line with our findings, it is possible to improve soft tissues as a result of the normal position of skeletal structures due to the activation of sutural growth with maxillary protraction in patients with a pseudo-Class III malocclusion, which is characterized by the insufficiency of soft tissues located in the upper lip and mid-face, by examining 3D images of patients with a Class III malocclusion. Improvements in soft tissues can also be achieved by limiting growth or guiding the growth of skeletal structures as the result of guiding the growth of condylar growth in true Class III patients, which is characterized by the prominence in soft tissues that include the lower lip and lower face.

## Conclusion

As a result of using 3dMD images to guide clinicians in the differential diagnosis of Class III malocclusions:

- Volume and linear measurements in the middle part of the face can be used. Midface volumes decreased significantly in pseudo-Class III malocclusions compared to both Class I and true Class III individuals.
- Chin volume can be measured in the differential diagnosis of skeletal Class III malocclusions. Chin volume increased significantly in skeletal Class III malocclusions compared to both Class I and pseudo-Class III individuals.
- Upper lip volume can be measured to distinguish Class I individuals from those with a pseudo-Class III malocclusion. Upper lip volumes were significantly higher in patients with Class I malocclusions than in those with a true or pseudo-Class III malocclusion.
- In skeletal and pseudo-Class III groups, mid-face measurements were significantly higher in males, while in the Class I malocclusion group, soft tissue characteristics were almost the same across the genders.

## Data Availability

All data generated and analyzed during this study were included in this manuscript.

## References

[1] Chong YH, Ive JC, Artun J (1996). Changes following the use of protraction headgear for early correction of Class III malocclusion. Angle Orthod..

[2] Ngan P, Moon W (2015). Evolution of Class III treatment in orthodontics. Am. J. Orthod. Dentofacial Orthop..

[3] Khan MB, Karra A (2014). Early treatment of class III malocclusion: a boon or a burden?. Int. J. Clin. Pediatr. Dent..

[4] Ngan PW, Sung JH, Nanda R (2014). Chapter treatment strategies for developing and nondeveloping Class III malocclusions. Esthetics and Biomechanics in Orthodontics.

[5] Williams S, Andersen CE (1986). The morphology of the potential Class III skeletal pattern in the young child. Am. J. Orthod..

[6] Guyer EC, Ellis EE, McNamara JA, Behrents RG (1986). Components of Class III malocclusion in juveniles and adolescents. Angle Orthod..

[7] Ngan P, Hu AM, Fields HW (1997). Treatment of Class III problems begins with differential diagnosis of anterior crossbites. Pediatr. Dent..

[8] Rabie AB, Gu Y (2000). Diagnostic criteria for pseudo-Class III malocclusion. Am. J. Orthod. Dentofacial Orthop..

[9] Moyers RE (1988). Handbook of Orthodontics.

[10] Major PW, Glover K (1992). Treatment of anterior crossbite in early mixed dentition. J. Can. Dent. Assoc..

[11] Lee BD (1978). Correction of crossbite. Dent. Clin. N. Am..

[12] Chen F, Terada K, Wu L, Saito I (2007). Dental arch widths andmandibularmaxillary base width in Class III malocclusions with low, average and high MP-SN angles. Angle Orthod..

[13] Nakasima A, Ichinose M, Nakata S (1986). Genetic and environmental factors in the development of so-called pseudo-and true mesioclusions. Am. J. Orthod. Dentofacial Orthop..

[14] Da Silveira AC, Daw JL, Kusnoto B (2003). Craniofacial applications of three-dimensional laser surface scanning. J. Craniofac. Surg..

[15] Alami, S., Aghoutan, H., Quars, F. E., Diouny, S. and Bourgui, F. Early treatment of anterior crossbite relating to functional class III. In *Emerging Trends in Oral Health Sciences and Dentistry*, p. 341 (2015).

[16] Coşkun Dİ, Kaya B (2012). Early orthodontic treatments. Turk. J. Orthod..

[17] Karatas OH, Toy E (2014). Three-dimensional imaging techniques: a literature review. Eur. J. Dent..

[18] Lane C, Harrell W (2008). Completing the 3-dimensional picture. Am. J. Orthod. Dentofac. Orthop..

[19] Hong C, Choi K, Kachroo Y, Kwon T, Nguyen A, McComb R, Moon W (2017). Evaluation of the 3dMD face system as a tool for soft tissue analysis. Orthod. Craniofac. Res..

[20] Baysal A, Sahan AO, Ozturk MA, Uysal T (2016). Reproducibility and reliability of three-dimensional soft tissue landmark identification using three-dimensional stereophotogrammetry. Angle Orthod..

[21] Dindaroğlu F, Kutlu P, Duran GS, Görgülü S, Aslan E (2016). Accuracy and reliability of 3D stereophotogrammetry: a comparison to direct anthropometry and 2D photogrammetry. Angle Orthod..

[22] Lowe DG (1987). Three-dimensional object recognition from single two-dimensional images. Artif. Intell..

[23] Chiu C, Clark R (1991). Reproducibility of natural head position. J Dent..

[24] Božič M, Kau CH, Richmond S, Ovsenik M, Hren NI (2010). Novel method of 3-dimensional soft-tissue analysis for Class III patients. Am. J. Orthod. Dentofac. Orthop..

[25] Martin O, Muelas L, Viñas MJ (2011). Comparative study of nasopharyngeal soft-tissue characteristics in patients with Class III malocclusion. Am. J. Orthod. Dentofac. Orthop..

[26] Kamak H, Celikoglu M (2012). Facial soft tissue thickness among skeletal malocclusions: is there a difference?. Korean J. Orthod..

[27] Jazmati HM, Ajaj MA, Hajeer MY (2016). Assessment of facial soft tissue dimensions in adult patients with different sagittal skeletal classes using cone beam computed tomography. J. Contemp. Dent. Pract..

[28] Devanna R, Althomali Y, Felemban NH, Manasali BS, Battepati PM (2017). Comparative evaluation of hard and soft tissue mid-face dimensions of Class I and Class III Individuals using CBCT. Indian J. Orthod. Dentofac. Res..

[29] Perovic T, Blažej Z (2018). Male and female characteristics of facial soft tissue thickness in different orthodontic malocclusions evaluated by cephalometric radiography. Med. Sci. Monit..

[30] Baccetti T, Franchi L, McNamara JA (2007). Growth in the untreated Class III subject. Semin. Orthod..

[31] Giovanoli P, Tzou CH, Ploner M (2003). Three-dimensional video-analysis of facial movements in healthy volunteers. Br. J. Plast. Surg..

[32] De Menezes M, Rosati R, Ferrario VF (2010). Accuracy and reproducibility of a 3-dimensional stereophotogrammetric imaging system. J. Oral Maxillofac. Surg..

[33] Moss J, Coombes A, Linney A (1991). Methods of three dimensional analysis of patients with asymmetry of the face. Proc. Finn. Dent. Soc..

[34] Singh GD, McNamara JA, Lozanoff S (1999). Finite-element morphometry of soft tissue morphology in subjects with untreated Class III malocclusions. Angle Orthod..

[35] Chang HP, Lin HC, Liu PH, Chang CH (2005). Midfacial and mandibular morphometry of children with Class II and Class III malocclusions. J. Oral Rehabil..

[36] Rabie AB, Gu Y (2000). Diagnostic criteria for pseudo-Class III malocclusion. Am. J. Orthod. Dentofac. Orthop..

[37] Spalj S, Mestrovic S, Lapter Varga M, Slaj M (2008). Skeletal components of class III malocclusions and compensation mechanisms. J. Oral Rehabil..

[38] Kılıç N, Oktay H, Çatal G, Çelikoğlu M (2018). Distinguishing hard and soft tissue facial morphology among class I and class III children: a cephalometric assessment. Atatürk Üniversitesi Diş Hekimliği Fakültesi Dergisi.

